# Characterization of Amyloid Cores in Prion Domains

**DOI:** 10.1038/srep34274

**Published:** 2016-09-30

**Authors:** Ricardo Sant’Anna, Maria Rosario Fernández, Cristina Batlle, Susanna Navarro, Natalia S. de Groot, Louise Serpell, Salvador Ventura

**Affiliations:** 1 Institut de Biotecnologia i Biomedicina and Departament de Bioquimica i Biologia Molecular, Universitat Autònoma de Barcelona, 08193 Bellaterra, Barcelona, Spain; 2 School of Life Sciences, University of Sussex, Falmer, BN1 9QG, UK

## Abstract

Amyloids consist of repetitions of a specific polypeptide chain in a regular cross-β-sheet conformation. Amyloid propensity is largely determined by the protein sequence, the aggregation process being nucleated by specific and short segments. Prions are special amyloids that become self-perpetuating after aggregation. Prions are responsible for neuropathology in mammals, but they can also be functional, as in yeast prions. The conversion of these last proteins to the prion state is driven by prion forming domains (PFDs), which are generally large, intrinsically disordered, enriched in glutamines/asparagines and depleted in hydrophobic residues. The self-assembly of PFDs has been thought to rely mostly on their particular amino acid composition, rather than on their sequence. Instead, we have recently proposed that specific amyloid-prone sequences within PFDs might be key to their prion behaviour. Here, we demonstrate experimentally the existence of these amyloid stretches inside the PFDs of the canonical Sup35, Swi1, Mot3 and Ure2 prions. These sequences self-assemble efficiently into highly ordered amyloid fibrils, that are functionally competent, being able to promote the PFD amyloid conversion *in vitro* and *in vivo*. Computational analyses indicate that these kind of amyloid stretches may act as typical nucleating signals in a number of different prion domains.

The connection between protein aggregation and human disease has fuelled the study of the molecular determinants underlying this process^1^. It is now clear that numerous and different kinds of proteins can build highly ordered amyloid aggregates, similar to those associated with disorders like Alzheimer’s or Parkinson’s^2^. Most amyloids share a common fibrillar structure constituted by repetitions of a specific polypeptide chain in a regular β-sheet rich conformation known as cross-β, owing to its X-ray diffraction pattern^3^. Despite the generic nature of protein aggregation, it is also clear that the propensity to self-assemble into amyloid fibrils is modulated by the specific protein sequence^4^,^5^. Importantly, whereas the folding of globular native states relies on interactions involving a large majority of the protein sequence^6^,^7^, amyloid formation seems to obey the “short-stretch hypothesis” in which a fibril is nucleated by specific and short sequence segments.

Prions constitute a subset of amyloids in which the aggregated state becomes self-perpetuating. The phenomenon is known mostly as a neuropathology in mammals, but in fungi, the aggregation of prions into amyloid-like conformations is exploited for beneficial purposes^8^,^9^,^10^. Despite the structural features shared between different amyloid aggregates, only a small set of them seems to behave as prions in natural environments. *Saccharomyces cerevisiae* prions are perhaps the best-characterized transmissible amyloids^11^. In these proteins, prion formation involves a structural conversion driven by relatively large, intrinsically disordered, domains enriched in glutamine/asparagine (Q/N) residues and, generally, depleted in hydrophobic and charged ones. Interestingly enough, protein domains displaying these signatures have been identified in over 250 human proteins, several of them linked to neurodegenerative diseases^12^.

The self-assembly of prion forming domains (PFDs) has been traditionally thought to ultimately rely on their particular amino acid composition^13^. According to this view, the prion-promoting residues are distributed across long unstructured segments from where they establish a large number of weak interactions^14^. The diffuse nature of the contacts proposed to drive prion aggregation and the consistent identification of PFDs in proteomes by composition-based computational methods^15^, has led to think that the “short-stretch hypothesis” does not apply to prions^16^. We have recently proposed that the presence and influence of specific amyloid-prone sequences within intrinsically disordered Q/N-rich regions are indeed important determinants of their prion behaviour^17^. These cores would differ, however, from those in disease-associated amyloids, where these regions generally comprise 5 to 10 residues, display a high aggregation propensity and possess a predominant hydrophobic character^18^. Because PFDs are intrinsically disordered, in case those highly aggregating regions would be present, they would be exposed to solvent and ready to aggregate, precluding prion proteins to remain in their soluble and functional conformation under physiological conditions. Accordingly, we have suggested that in PFDs, the amyloid stretches should be longer and less hydrophobic, in such a way that the amyloid potential is less concentrated, making their aggregation sensitive to protein concentration and seeding^17^. They should cluster in the same sequence region amino acid residues with a significant amyloid propensity with residues promoting structural disorder. Q and N, are the residues that best balance amyloid and disorder propensity^17^, which would explain the over-representation of these particular residues in PFDs in general and in their predicted amyloid cores in particular^17^. We have shown that the implementation of this concept in our in house pWALTZ algorithm allows classifying Q/N rich sequences according to their prionic behaviour with better accuracy than methods relying only on composition^17^,^19^. pWALTZ aims to predict the 21 residues long sequence stretch with the average highest amyloidogenic potential in a Q/N rich sequential context, a length that has been shown to suffice for the formation of transmissible β-folds in the case of the HET-s prion domain^20^ and corresponding to minimal core size allowing maximal discrimination between prionic and non-prionic sequences bearing similar Q/N content^17^.

Here, we provide experimental evidence for the existence of such Q/N rich amyloid stretches in the PFDs of four of the best-characterized yeast prion proteins: Sup35, Swi1, Mot3 and Ure2. pWALTZ identified potential, previously uncharacterized, 21 residues long amyloid stretches in the PFDs of the four proteins. The analysis of the corresponding peptides indicates that all them readily self-assemble into Th-T positive, β-sheet enriched, highly ordered amyloid fibrils displaying a typical cross-β diffraction pattern. Moreover, we demonstrate that the fibrils formed by the amyloid stretch of Sup35 are able to seed amyloid formation by its entire PFD with higher efficiency than the fibrils of the complete domain themselves. In addition, we show that infection of yeast cells with preformed peptide fibrils can promote the conversion of the endogenous Sup35 protein towards the prionic state. Finally, computational analysis indicates that mutations increasing prion propensity are often associated with the formation of more potent amyloid stretches in PFDs. Overall, this suggests that these relatively short sequences may act as important amyloid nucleating signals in prion domains.

## Results

### Identification of amyloid cores in Yeast Prion Domains

For the present study, we choose four of the best characterized prion proteins: Mot3, Sup35, Swi1 and Ure2^21^,^22^,^23^,^24^. All four are yeast proteins that execute essential regulatory functions required for cell adaptation and survival in different conditions. Sup35 is part of the translation termination complex that regulates stop-codons read-through. In the soluble form, the complex binds the stop-codon and ends the translation; however, in the prion form [PSI^+^] the translation continues, increasing genetic variation and the chances of survival in changeable environments^25^,^26^. Swi1 is a chromatin-remodelling factor that regulates the expression of 6% of the yeast genome^23^, its self-assembly causes an expression pattern change that can be beneficial under certain conditions^27^. Mot3 is a transcription factor that regulates the expression of several hypoxic genes. Under aerobic conditions the soluble form represses, whereas under anaerobic conditions the prion form [MOT3^+^] releases the expression of these genes^24^. Ure2 is a nitrogen metabolism regulator. In rich nitrogen conditions, the soluble form represses the use of alternative nitrogen sources, but when nitrogen is limiting, Ure2 self-assembles stopping this repression^28^. Therefore, the switch between the prion and soluble forms of these proteins is crucial for cell adaptation and so it is the control and regulation of this phenomenon.

**Figure 1:**
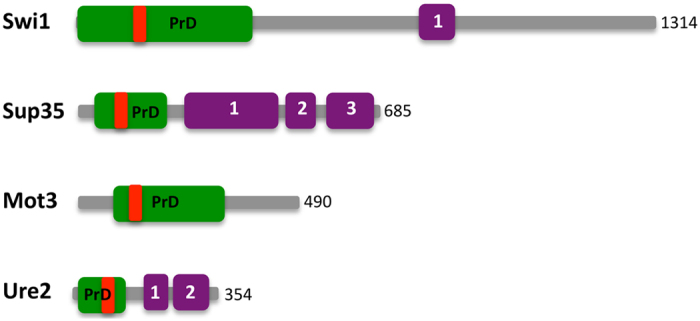
The four proteins studied here are represented with their respective known functional domains (lilac). Swi1: (1) ARID/BRIGHT DNA binding domain; Sup35: (1) Elongation factor Tu GTP binding domain, (2) Elongation factor Tu domain 2 and (3) Elongation factor Tu C-terminal domain; Ure2: (1) Glutathione S-transferase N-terminal domain and (2) Glutathione S-transferase C-terminal domain. Prion domains are shown in green and the predicted 21 residues amyloid core inside the prion domain in red. The functional domains were assigned according to Pfam (http://pfam.xfam.org) and the PFD according to UniProt (http://uniprot.org). The exact position of the amyloid cores in the PFDs and their amino acid sequences are displayed in Table 1.

Computational analyses have led us to propose that PFDs may contain specific amino acid regions capable of nucleating the self-assembly process^17^. To validate this view, we analysed the presence of potential amyloid forming sequences within the PFDs of the four prions using pWALTZ^17^. The selected PFDs boundaries were those annotated in UniProt (http://www.uniprot.org) (Table 1). In all cases we could predict the presence of an amyloid core embedded in the corresponding PFD (Fig. 1 and Table 1). In contrast, conventional aggregation predictors like TANGO^29^, AGGRESCAN^30^ or PASTA^31^ failed to predict any significant amyloid propensity in any of the four pWALTZ identified sequence stretches, since they are intended to identify shorter stretches with more concentrated aggregation potential and the polar Q and N residues score low in these algorithms (data not shown).

### Predicted PFDs amyloid cores assemble into β-sheet rich structures

The aggregation of proteins and peptides into amyloid fibrils usually results in the formation of intermolecular β–sheets^32^. We tested if the 21 residues-long peptides corresponding to the four PFDs amyloid cores were able to convert their initially soluble state into a β-sheet rich aggregated conformation using both Far-UV circular dichroism (CD) and infrared spectroscopy (ATR-FTIR) to monitor the changes in their secondary structure content upon incubation of the peptides for 2 h at 100 μM in PBS at pH 7.4.

**Figure 2:**
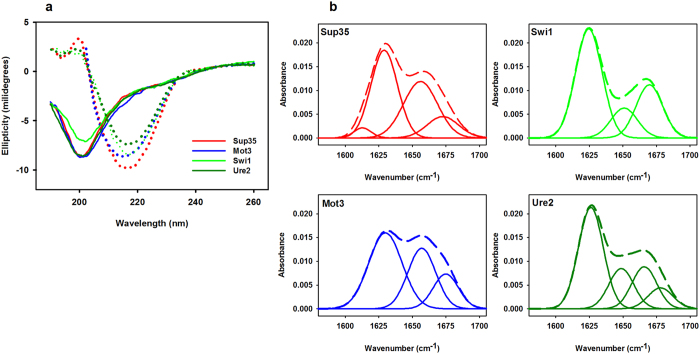
PDFs amyloid cores secondary structure. The peptides secondary structure was analyzed by Far-UV CD (**a**) before (solid lines) and after incubation (dotted lines). The initial characteristic 200 nm negative peak corresponding to disordered structure displaces to 216 nm, indicative of a β-sheet content, upon incubation. The secondary structure of the incubated peptides was also analysed by ATR-FTIR (**b**). All of them exhibit a major band at ∼1625 cm^−1^ indicating the presence of inter-molecular β-sheets. Additional secondary structure components are detailed in Table 2.

The comparison of the CD spectra before and after incubation (Fig. 2a) shows that, in all cases, the spectra minimum shifts from 200 nm to 216 nm. This is consistent with the peptides switching from an initially disordered, random coil structure, to a β-sheet enriched conformation. To further characterize this transition, we analyzed the amide I region of the FTIR spectrum (1700–1600 cm^−1^), which corresponds to the absorption of the carbonyl peptide bond group of the protein main chain. Deconvolution of the FTIR spectra allowed us to assign the individual secondary structure elements of incubated peptides and their relative contribution to the main absorbance signal (Fig. 2b and Table 2). In all cases, a band at ∼1625 cm^−1^ dominates the spectrum. This spectral component is usually attributed to the presence of intermolecular β–sheet structure and accounts for 50–60% of the area of the peptides spectrum area. Interestingly, no anti-parallel β–sheet band was detected (~1690 cm^−1^)^33^, suggesting thus that the β–strands in self-assembled peptides would adopt a parallel disposition. The other detected structural elements are associated with a disordered structure, loops and β–turns (Table 2). Overall, the CD and FTIR data are consistent with the assembly of the predicted PFDs amyloid cores into a highly enriched β–sheet structure.

### Predicted PFDs amyloid cores assemble into amyloid fibrils displaying a cross-β structure

**Figure 3:**
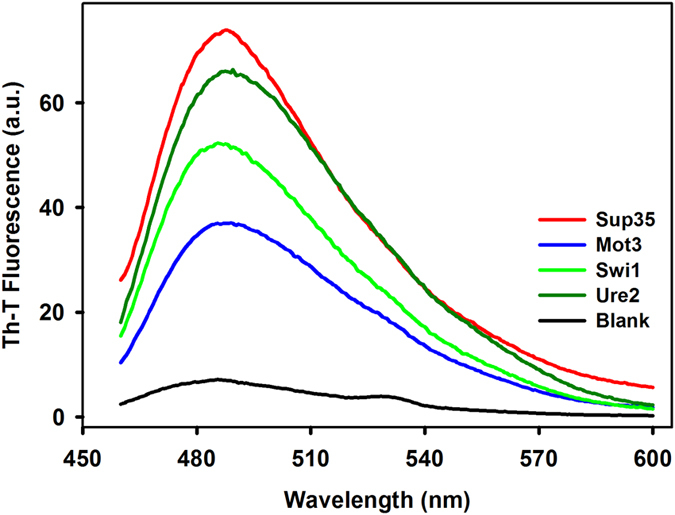
Thioflavin-T binding to PDFs amyloid cores. The peptides were let to aggregate in quiescent solutions for 2 hours at RT and 100 μM. Then, 10 μL of soluble or aggregated samples were mixed with a solution of Th-T at 30 μM and fluorescence was measured by exciting the samples at 450 nm and collecting their emission from 460 to 600 nm.

We used the amyloid-specific dye Thioflavin T (Th-T) to confirm that the detected β–sheet assemblies in incubated peptides were organized into amyloid-like superstructures. Th-T fluorescence emission is enhanced in the presence of amyloid fibrils^34^,^35^. Whereas incubation of Th-T with the initially soluble peptides did not promote any detectable spectral change, when incubated for 2 h, a large increase in Th-T fluorescence emission intensity was observed for all peptide solutions (Fig. 3).

**Figure 4:**
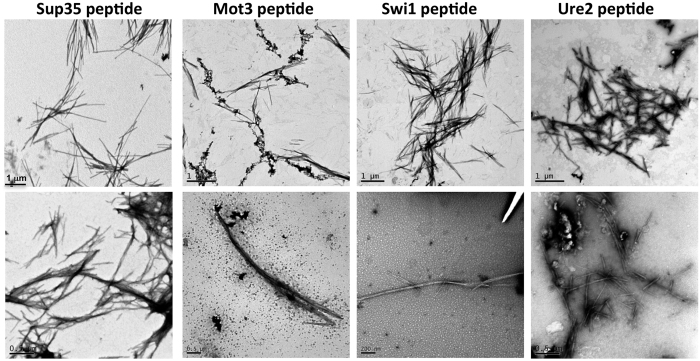
Fibrillar structure of Sup35, Mot3, Swi1 and Ure2 PFDs amyloid cores. On the upper images bars corresponds to 1 μm and on the lower images to 0.5 μm for Sup35, Mot3 and Ure2 and to 0.2 μm for Swi1.

To confirm that Th-T binding reported on amyloid-like structure, the morphological features of the peptides assemblies were analysed using transmission electron microscopy (TEM). Negative staining indicated that all peptides self-assemble forming fibrillar arrangements displaying the basic characteristics of amyloid fibrils, since they appear straight and unbranched, with a tendency to coalesce laterally (Fig. 4). The fibrils exhibit a diameter that varies from 5 to 10 nm and a length that ranges from 2 to 10 μm.

**Figure 5:**
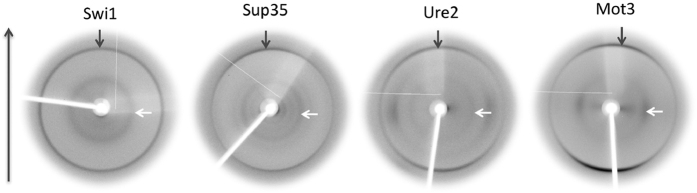
X-ray diffraction patterns collected from partially aligned fibril samples formed by the PFDs. Fiber axis is vertical and the arrows highlight the characteristic “cross-β” reflections. Grey 4.76 Å meridional, white 9–11 Å equatorial reflections.

X-ray diffraction experiments were performed on fibers containing peptide amyloid fibrils. The X-ray diffraction patterns indicate that amyloid fibrils formed in every case possess the typical ‘cross-β’-like architecture (Fig. 5). Specifically, a strong meridional diffraction signal is evident at 4.6–4.7 Å which arises from the repeat along the axis of the fibers, and represents the distance between successively hydrogen bonded β-strands which are aligned perpendicular to the fibril axis. Moreover, all patterns exhibit a second strong reflection along the equator, corresponding to a d-spacing of 8.5–9.7 Å. This structural repeat is likely to arise from the packing distance between β-sheets that are aligned parallel to the fibril axis (Table S1). The differences observed in the equatorial reflections indicate dissimilarities in the packing distance of each peptide and likely arise from the variable sizes of the side chains that are interlocked in the steric-zippers formed between the β-sheets in each case.

### Sup35 PFD amyloid core seeds Sup35-NM domain amyloid formation *in vitro*

The assays reported above demonstrate that the predicted amyloid cores are able to self-assemble into amyloid fibrils. However, these data alone do not prove that these sequences would be able to recruit and nucleate the arrangement of a full-length disordered PFD into an amyloid structure. To test this possibility, we analysed if peptide amyloid fibrils were able to accelerate (seed) the aggregation reaction of the entire PFD in the case of the Sup35 protein.

**Figure 6:**
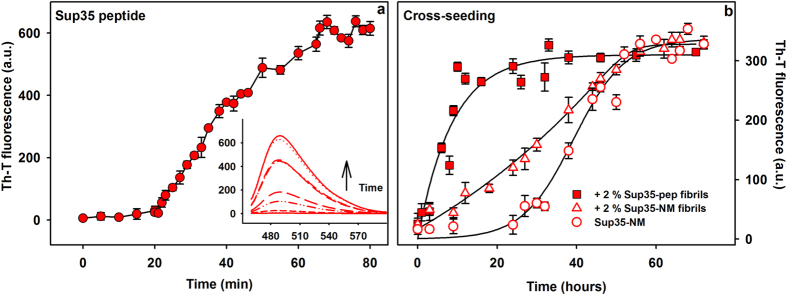
Aggregation kinetics, seeding and cross-seeding reactions of Sup35 PFD amyloid core and Sup35-NM domain. In panel a the aggregation kinetics were carried out by incubating 100 μM of peptide with 30 μM of Th-T at RT with no agitation and the fluorescence measured along time. Th-T spectra were collected by exciting the samples at 450 nm and collecting the emission from 460 to 600 nm. The intensity at 482 nm was used to monitor the extent of aggregation. In the inset are shown some representative spectra. In panel b, the Sup35-NM domain was incubated at 10 μM, at RT, under agitation of 400 rpm in the absence of seeds or self-seeded with 2% of its own fibrils or seeded with 2% of the fibrils formed by the Sup35 PFD peptide. The three reactions were carried out simultaneously and monitored by Th-T fluorescence. At each time point, a 10 μL aliquot of each sample was mixed with 30 μM of Th-T solution and the fluorescence spectra collected. The intensity at 482 nm was used as a proxy of fibril formation.

We performed self and cross-seeding assays between the Sup35 peptide and the Sup35-NM prion domain (residues 1–253). The N-terminal region comprises the PFD (residues 1–114), which is defined as the minimal region essential for induction and propagation of the prion state^36^. Acting as a linker between the N and the elongation factor Tu GTP binding domain of Sup35, the highly charged M region increases the solubility of the protein^37^ and imparts stability to the prion during mitosis and meiosis^38^. The aggregation kinetics of the peptide and Sup35-NM were measured by monitoring the changes in Th-T fluorescence (Fig. 6). The peptide kinetics corresponds to a characteristic sigmoidal aggregation reaction with a short lag phase of 20 min, reaching the stationary phase in less than 90 min, indicative of a fast self-assembly potential (Fig. 6A). In contrast, the aggregation of Sup35-NM is much slower, displaying a lag phase that lasts for 20 h, being completed only after 50 h of reaction (Fig. 6B). The addition of 2% preformed Sup35-NM seeds significantly shortens the lag phase, supporting a nuclei-dependent aggregation mechanism^39^, however the Th-T signal increases slowly achieving its maximum intensity also at ~50 h. In contrast, the addition of 2% peptide amyloid seeds results in a fast and hyperbolic increase in Th-T signal that achieves its maximum intensity at 22 h without any detectable lag phase. Therefore, the conversion of soluble Sup35-NM into the amyloid state is at least two times faster when it is initiated by the amyloid core than by the complete domain itself. We also analysed whether preformed amyloid fibrils of Sup35, Ure2, Mot3 and Swi1 peptides were able to seed the aggregation of initially soluble Sup35 amyloid core. No cross-seeding effect was detected and aggregation could be only accelerated by the fibrils of the same Sup35 peptide, which abrogated the lag phase of the reaction (Figure S1), thus confirming sequence specificity in PFDs aggregation.

### Sup35 amyloid core can promote endogenous Sup35 prion conversion in yeast

**Figure 7:**
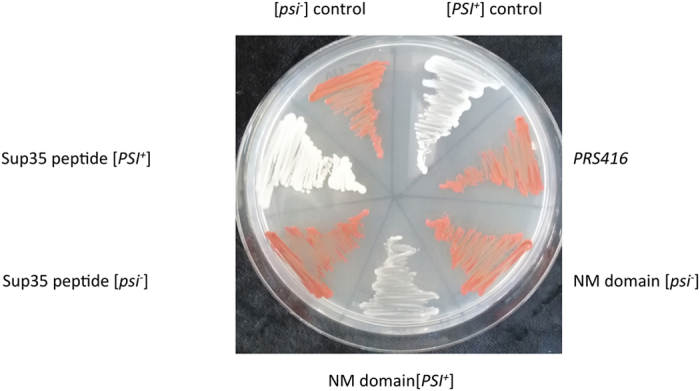
Conversion of non prionic [psi^−^] to prionic [PSI^+^] yeasts by transformation with Sup35 PFD amyloid core peptide or the complete Sup35-NM domain. Yeast cells (L1749) were transformed by incubation of spheroplasted cells with the transformation mixtures, containing either 10 μg/mL pRS416 vector, or the pRS416 vector plus 40 μM of Sup35 peptide fibrils, or pRS416 plus 40 μM of NM-domain fibrils. 100 colonies were plated and screened for PSI positive and negative prion phenotypes in ¼ YPD plates. Representative positive and negative colonies are shown in this figure, together with the positive and negative control strains: L1749 [psi^−^] and L1762 [PSI^+^].

The amyloid fibrils formed *in vitro* by the Sup35-NM domain have been shown to be infectious if they enter into yeast cells. They induce the conversion of cells displaying the [psi^−^] phenotype into the prionic [PSI^+^] state, by seeding the conversion of the originally soluble native Sup35 protein into insoluble amyloid structures^40^. Provided that Sup35 PFD amyloid core fibrils sufficed to seed the aggregation of Sup35-NM *in vitro,* we explored if they were also able to promote the conversion of [psi^−^] yeast cells into [PSI^+^] ones. Purified Sup35-NM and Sup35 amyloid core fibrils and a vehicle solution were used to transform spheroplasts of a [psi^−^] yeast strain as described in the Material and Methods section. Subsequently, they were streaked in ¼ YPD plates. On these plates, [psi^−^] cells are of an intense red color, whereas [PSI^+^] cells appear white^41^. No [PSI^+^] colonies were observed for transformations with vehicle alone. In contrast, transformation with recombinant Sup35-NM and synthetic Sup35 PFDs amyloid core fibrils resulted in a 12% and 4% of [PSI^+^] colonies, respectively (Fig. 7), indicating that both assemblies are able to interact with and promote the aggregation of native endogenous Sup35 within the cell.

### Mutations in amyloid cores impact prion propensity

For classical amyloids, the existence of amyloid cores explains why mutations that increase the aggregation potential of pre-existent sequence stretches or that create a novel aggregation-prone region usually result in more aggressive or early onset pathological phenotypes^42^,^43^. By analogy, one would expect that mutations in the PFDs promoting the same effects would increase their prionogenicity. Indeed, we have previously observed this effect when analysing mutations of human hnRNPA1 and hnRNPA2 prion-like proteins associated with multisystem proteinopathy and amyotrophic lateral sclerosis. The pathogenic mutations occur in the PFDs of hnRNPA1 and hnRNPA2 and map precisely in the 21-residues long region identified by pWALTZ, increasing in all cases the predicted amyloidogenicity of these sequence stretches^17^,^44^. In a recent work, Ross and co-workers have used targeted mutation to convert four prion-like domains having high compositional similarity to yeast prions, but devoid of prion activity, into functional PFDs^45^. The analysis of these sequences with pWALTZ indicates that, in all cases, those mutations resulting in novel prionic activity have created a short sequence stretch with a higher amyloid propensity than the original one (Table 3). These data suggest that, similar to what happens in classical amyloid stretches, if a mutation increases the amyloid potential of a nucleating region in a PFD it would favour sequence specific self-assembly and thus the population of aggregated prionogenic states.

## Discussion

The formation of amyloid assemblies is connected to a significant number of human disorders^1^. Many research efforts have been directed towards understanding the factors underlying this process because they hold the key for the development of novel therapeutic strategies. One of the outcomes of these efforts is the so-called “short-stretch hypothesis”, which postulates that a short amino acid stretch could provide most of the driving force needed to trigger the self-assembly of a protein into an amyloid^46^,^47^,^48^. This observation has pushed the development of over twenty different algorithms, aimed to identify these sequence stretches in disease-linked polypeptides^49^,^50^, whose predictions have been experimentally validated in many instances. Indeed, compounds blocking these regions have been shown to successfully interfere with entire protein aggregation reactions^51^ and conversely mutations that increase the amyloid propensity of these stretches usually exacerbate full-length protein deposition^52^. There was debate on whether the driving force of the amyloid self-assembly was the composition of these stretches or their sequence. By reversing and scrambling an amyloid sequence stretch we provided evidence that it is the sequence that governs the assembly pathway and the final amyloid conformation^53^. This explains why cross-aggregation often results in the formation of disordered aggregates and why cross-seeding reactions between proteins differing in their sequence are rare^54^.

Despite the morphological similarities between the aggregates formed by disease-linked amyloids and the Q/N rich functional yeast prions, at least *in vitro*, conventional β-aggregation and amyloid predictors fail to identify and score the last type of proteins. This inaccuracy owes essentially to the fact that yeast PFDs are constituted by intrinsically disordered segments, which, by definition are, poor in hydrophobic residues and rich in polar residues. Accordingly, it has been assumed that the transition of PFDs from the soluble state to the amyloidogenic one arises from a bias in sequence composition favouring the establishment of a large number of weak interactions along the entire PDF segment^55^. However, we have recently proposed that prion behaviour also emerges from the preferential nucleation by specific and localized amyloid-prone stretches embedded in the wider disordered region^17^,^19^. Here we provide experimental evidence for the presence of these regions in the PFDs of four different yeast prions and for the ability of one of these amyloid cores to recruit the organized assembly of its corresponding full-length prion domain *in vitro* and inside the cell. This suggests that the ‘classical’ short-stretch nucleation model applies to prions in a similar manner as it does for the rest of amyloids. Thus, the main differences between these two protein types are that in prions the amyloid nucleating potency is weaker and less concentrated, being spread in a larger segment. This property allows the protein to remain soluble in most physiological conditions, but also to respond to the presence of preformed seeds that target these segments and promote the prion self-assembly. This short-stretch nucleation mechanism would permit a more precise control of self-assembly and transmissibility^12^ than the establishment of several weak interactions through a long disordered and presumably highly flexible PFD, which will imply a high entropic cost already at the beginning of the reaction. In a way, the initial self-assembly step in PFDs would resemble to that mediated by short lineal motifs (SLIMs) residing in disordered regions, this location makes them accessible to mediate protein-protein interactions roughly independently from the rest of the polypeptide^56^. The length of 21-residues was selected because it provided the best discrimination between prionic and non-prioninc Q/N rich domains^17^, but of course it is arbitrary and amyloid cores may have different lengths in different PFDs, also, we are not arguing that the identified amyloid cores are the only stretches that can or are necessary for prion conversion and, as it happens in classical amyloids, other short regions can also cooperate in the assembly reaction.

Selective pressure to reduce the burden of protein aggregation in biological organisms has minimized the aggregation propensities of protein sequences^57^, especially in intrinsically disordered protein segments where they are exposed to solvent^58^. In this context, the significant amyloid potential of the assayed sequences suggests that they are conserved because they serve functional purposes, being likely involved in the self-organization of prion macromolecular assemblies in living cells^59^.

## Materials and Methods

### Sup35-NM overexpression and purification

20 ml of LB (100 μg/ml Amp, 34 μg/ml Clm) were inoculated with single colonies of BL21(DE3) pLysS cells transformed with a pET plasmid encoding for Sup35-NM (residues 1 to 254) and incubated overnight at 37 °C and 250 rpm. Subsequently, the culture was transferred into 2 L LB/Amp/Clm and incubated at 37 °C and 250 rpm. Protein expression was induced at OD_600_ = 0.6 by addition of isopropyl-*β*-D-thiogalactopyranoside (IPTG) to a final concentration of 1 mM and the culture left O/N. Cells were then harvested by centrifugation for 20 min at 7000 g (5296 rpm) and 4 °C (Beckman CoulterTM Avanti Centrifuge J-26XPI). The cell pellet was resuspended in 50 ml of denaturing buffer (20 mM Tris pH 8, 0.5 M NaCl, 6 M guanidine, 20 mM imidazole). The suspension was sonicated for 10 min on ice (Branson Digital Sonifier). The lysate was centrifuged for 45 min at 48384 g and 4 °C. The supernatant was sonicated again for 10 min and then filtered using a 0.45 μm low binding Millipore filter. A Nickel column HisTrap FF of 5 ml (GE Healthcare) was used to purify Sup35-NM carrying a C-terminal polyhistidine-tag. Sup35-NM was eluted from the column by one-step with 25 ml of elution buffer (20 mM Tris pH 8, 0.5 M NaCl, 6 M guanidine, 500 mM imidazole). The protein was further concentrated using an Amicon Ultra Centrifugal Filter (Merck KGaA).

### Peptides prediction and synthesis

The sequences of the PFDs of Sup35 (P05453), Swi1 (P09547), Mot3 (P54785) and Ure2 (P23202) proteins as annotated in UniProt were analyzed with pWALTZ^17^. Twenty-one residues-long peptides corresponding to the respective predicted amyloid cores (Table 1) were purchased from CASLO ApS (Scion Denmark Technical University). The peptides were weighted and dissolved in DMSO to obtain a stock solution of 10 mM. For Circular Dichroism analysis the stock solutions were prepared in TFE at 10 mM to avoid the intense background noise caused by residual DMSO on the CD spectra.

### Peptides Aggregation Assays

Right before each experiment the stock solutions were diluted to 100 μM in PBS pH 7.4. To ensure reproducibility in the aggregation reactions, the diluted solutions were filtered 2 times through a low protein binding 0.22 μm Millipore filter right before starting the measurements. The peptide containing solutions were incubated at room temperature for 2 h on a ST-5 horizontal agitator (CAT Ingenieurbüro M. Zipperer GmbH) with shaking set to 60 rpm/min. To detect the formation of amyloid aggregates, Th-T was mixed with the sample to a final concentration of 30 μM and its fluorescence measured. Samples were excited at 450 nm while emission was collected from 460 to 600 nm. The aggregation kinetics of the Sup35 peptide was followed by monitoring the change in Th-T fluorescence intensity at 482 nm along time.

### Circular dichroism

Circular dichroism (CD) was used to determine the main secondary structure presented by soluble and aggregated peptides at the times stated on the experiments. For the soluble state, the measurement was performed right after diluting the peptide stock to 20 μM in PBS. For measurements in the aggregated state, the same samples were measured again after two hours of incubation. Spectra were recorded between 200–250 nm, at a resolution of 1 cm^−1^, with a 15 nm·min^−1^ scan rate and at room temperature in a J-810 spectropolarimeter (Jasco Corp. Tokyo, Japan) using a quartz cell of 0.1 cm path length.

### Infrared spectroscopy

Attenuated total reflectance Fourier transform infrared spectroscopy (ATR FT-IR) was used to determine the secondary structure content of the amyloid fibrils formed by the peptides. After the aggregation time (2 h), the samples were sonicated and 3 μL placed on the ATR crystal for measurements. Samples were dried under a N_2_ stream. The experiments were carried out using a Bruker Tensor 27 FT-IR spectrometer (Bruker Optics) with a Golden Gate MKII ATR accessory. Each spectrum consists of 16 accumulations measured at a resolution of 2 cm^−1^ in a wavelength range between 1700 and 1600 cm^−1^. Infrared spectra were fitted through overlapping Gaussian curves, and the amplitude, mass center, bandwidth at half of the maximum amplitude, and area for each Gaussian function were calculated employing a nonlinear peak-fitting equation using PeakFit package (Systat Software, San Jose, CA).

### Transmission Electron Microscopy

Aggregated samples were diluted in Milli Q water to a final concentration of 10 μM. 5 μl of the solution were absorbed onto carbon-coated copper grids for 5 minutes and blotted to remove excess material. Uranyl acetate (2% w/v) was used for negative staining. Samples were dried on air for 5 minutes. Grids were exhaustively scanned with a Hitachi H-7000 transmission electron microscope operating at a voltage of 75 kV.

### X-Ray diffraction

The aggregates were grown in the conditions described above. The fibrils were isolated by centrifugation at 13,400 rpm for 30 minutes. The pellets were washed two times in Milli Q water to the discard of any residue of salt and the samples were concentrated to 10 mg/mL protein concentration.

Fibril samples were aligned between wax-tipped capillaries and allowed to air dry^60^. The partially aligned fiber was placed on a goniometer head and data were collected using Rigaku rotating anode source (CuKα) and Saturn 944 + CCD detector. The diffraction patterns were examined with Mosflm (CCP4)^61^ and then output as TIFs. Measurements of the signal positions were conducted using Clearer^62^.

### Sup35-NM seeding assay

The stock solution of Sup35-NM domain was maintained in Guanidine 6 M to avoid any residual initial aggregation. At the beginning of the experiment, the protein was diluted to a final concentration of 10 μM in phosphate buffer (5 mM potassium phosphate, 150 mM NaCl, pH 7.4). Reactions were carried out at RT in the absence or presence of seeds. The seeds were prepared by sonicating the preformed fibrils of the Sup35 PFD amyloid core peptide or the fibrils of Sup35-NM for 10 min. The fibrils of the Sup35-NM domain were formed essentially as described^63^. Briefly, 10 μM of protein were to aggregate without agitation at RT for at least 5 days. The amount of seeds used was 2% (v/v). At different time points, 10 μL of each sample were mixed with Th-T (30 μM) and the fluorescence emission intensity at 482 measured.

### Sup35-NM domain and Sup35 peptide transformation and analysis of phenotypes

Yeast strain L1749 (*MAT α*, *ade1-14, ura3-52, leu2-3,112, trp1-289, his3-200,* [*psi*^−^], [*PIN*^*+*^]) was transformed by the method previously described^64^ with some modifications. Briefly, yeast cells were grown on YPD (1% yeast extract, 2% peptone, 2% glucose) to OD_600_ ≈ 1.0. Yeast cells were successively washed with sterile water, 1 M sorbitol, and finally resuspended in SCE buffer (1 M sorbitol, 10 mM EDTA, 10 mM DTT, 100 mM sodium citrate, pH 5.8). Cell wall was removed by lyticase treatment, and the resulting spheroplast cells were transformed with either the vector pRS416 in 2.2% DMSO (negative control), Sup35 peptide plus pRS416 in 2.2% DMSO, or Sup35-NM domain plus pRS416 in 2.2% DMSO. Previously to the transformation, Sup35 peptide and Sup35-NM were seeded for fibrillation, and sonicated for 20 min in a water-bath sonicator immediately before the transformation. Yeast spheroplast were washed with 1 M sorbitol and resuspended in STC buffer (1 M sorbitol, 10 mM Tris-HCl, 30 mM CaCl_2_, pH 7.5). Transformation was carried out by incubation of spheroplasted cells with the previous mixtures of vector, vector plus peptide or NM-domain, in the presence 200 *μ*g/mL ssDNA and PEG solution (20% PEG8000, 30 mM CaCl_2_, 10 mM Tris-HCl, pH 7.5). Yeast cells were resuspended in SOS medium (1 M sorbitol, 6.5 mM CaCl_2_, 0.25% yeast extract and 0.5% bacto peptone), and incubated at 28 °C for 30–45 min. Cells were mixed with top-agar (1 M sorbitol, 2.5% bacto agar) and plated on selective medium SD-Ura (0.17% yeast nitrogen base without ammonium and amino acids, 0.5% ammonium sulfate, 2% glucose, 2% bacto agar, supplemented with amino acids but without uracil). After incubating plates at 28 °C during for 3–5 days, between 50–100 transformants were randomly selected, streaked onto ¼ YPD plates and incubated at 28 °C for 3 days.

## Additional Information

**How to cite this article**: Sant’Anna, R. *et al.* Characterization of Amyloid Cores in Prion Domains. *Sci. Rep.* **6**, 34274; doi: 10.1038/srep34274 (2016).

## Supplementary Material

Supplementary Information

## Figures and Tables

**Table 1:** Yeast prion forming domains boundaries, predicted amyloid cores, pWALTZ scores and Q/N content.

Protein	UniProt Id (PFD)	Amyloid Core	pWALTZ score	Q/N (%)
Sup35	P05453 (5–135)	98-RGNYKNFNYNNNLQGYQAGFQ-118	73.99	43
Mot3	P54785 (98–295)	123-NSNNSNISASDYTVANNSTSN-143	74.27	33
Swi1	P09547 (1–323)	239-FNNSASNNGNLTSNQLISNYA-259	75.42	38
Ure2	P23202 (2–89)	20 -NIGNRNSNTTTDQSNINFEFS-40	73.65	29

**Table 2:** Assignment and area of the secondary structure components of aggregated PFDs amyloid cores in the amide I region of FTIR spectra.

Assignments	Sup35	Mot3	Swi1	Ure2
Inter β-sheet (1623–1641 cm^−1^)	59%	49%	57%	50%
Disordered (1642–1647 cm^−1^)	25%	32%	16%	19%
Turns (1662–1686 cm^−1^)	16%	—	27%	21%
β-sheet (1674–1695 cm^−1^)	—	19%	—	10%

**Table 3:** Yeast PFDs mutants displaying increased prion propensities display a stronger amyloid core.

Protein	PFD amyloid core	pWaltz Score
Puf4	HGYYNNNNNNNNNNNNNNNSN	73.8493
Puf4^mut^	FNNNSMSSNNINVNIGNSN**YN**	74.2634
YL177	–––––––––––––	<35.00
YL177^mut^	QQQSIF**YQ**G**NV**SSYIS**N**ALIH	76.1426
KC11	LQMQQLQMQQLQQQQQQQQYA	64.0547
KC11^mut^	QQQQYAQ**Y**T**N**A**F**M**I**NSQY**VYN**	82.6883
PDC2	GSSNNINDNDSSVKYLQQNTV	68.6574
PDC2^mut^	HN**NQ**YNSN**N**SNNAI**F**NTNN**Y**G	77.1778

Amyloid cores of wild type and mutant (^mut^) PFDs and their respective pWALTZ scores are shown. Mutated residues in the PFDs overlapping with the predicted amyloid core are shown in bold.

## References

[b1] Chiti F. & Dobson C. M. Protein misfolding, functional amyloid, and human disease. Annu. Rev. Biochem. 75, 333–366 (2006). 16756495 10.1146/annurev.biochem.75.101304.123901

[b2] Goldschmidt L., Teng P. K., Riek R. & Eisenberg D. Identifying the amylome, proteins capable of forming amyloid-like fibrils. Proc. Natl. Acad. Sci. USA 107, 3487–3492 (2010). 20133726 10.1073/pnas.0915166107 PMC2840437

[b3] Sabate R. & Ventura S. Cross-beta-sheet supersecondary structure in amyloid folds: techniques for detection and characterization. Methods Mol. Biol. 932, 237–257 (2013). 22987357 10.1007/978-1-62703-065-6_15

[b4] Linding R., Schymkowitz J., Rousseau F., Diella F. & Serrano L. A comparative study of the relationship between protein structure and beta-aggregation in globular and intrinsically disordered proteins. J. Mol. Biol. 342, 345–353 (2004). 15313629 10.1016/j.jmb.2004.06.088

[b5] Bemporad F. *et al.* Sequence and structural determinants of amyloid fibril formation. Acc. Chem. Res. 39, 620–627 (2006). 16981678 10.1021/ar050067x

[b6] Anfinsen C. B. Principles that govern the folding of protein chains. Science 181, 223–230 (1973). 4124164 10.1126/science.181.4096.223

[b7] Fass D. Disulfide bonding in protein biophysics. Annual review of biophysics 41, 63–79 (2012). 10.1146/annurev-biophys-050511-102321 22224600

[b8] Aguzzi A. & Calella A. M. Prions: protein aggregation and infectious diseases. Physiol Rev 89, 1105–1152 (2009). 19789378 10.1152/physrev.00006.2009

[b9] Chien P., Weissman J. S. & DePace A. H. Emerging principles of conformation-based prion inheritance. Annu. Rev. Biochem. 73, 617–656 (2004). 15189155 10.1146/annurev.biochem.72.121801.161837

[b10] Sabate R. When amyloids become prions. Prion 8, 233–239 (2014). 24831240 10.4161/19336896.2014.968464 PMC4601197

[b11] Wickner R. B., Edskes H. K., Gorkovskiy A., Bezsonov E. E. & Stroobant E. E. Yeast and Fungal Prions: Amyloid-Handling Systems, Amyloid Structure, and Prion Biology. Adv. Genet. 93, 191–236 (2016). 26915272 10.1016/bs.adgen.2015.12.003 PMC9432818

[b12] King O. D., Gitler A. D. & Shorter J. The tip of the iceberg: RNA-binding proteins with prion-like domains in neurodegenerative disease. Brain Res. 1462, 61–80 (2012). 22445064 10.1016/j.brainres.2012.01.016 PMC3372647

[b13] Ross E. D., Edskes H. K., Terry M. J. & Wickner R. B. Primary sequence independence for prion formation. Proc. Natl. Acad. Sci. USA 102, 12825–12830 (2005). 16123127 10.1073/pnas.0506136102 PMC1200301

[b14] Toombs J. A., McCarty B. R. & Ross E. D. Compositional determinants of prion formation in yeast. Mol. Cell. Biol. 30, 319–332 (2010). 19884345 10.1128/MCB.01140-09 PMC2798286

[b15] Cascarina S. M. & Ross E. D. Yeast prions and human prion-like proteins: sequence features and prediction methods. Cell. Mol. Life Sci. 71, 2047–2063 (2014). 24390581 10.1007/s00018-013-1543-6 PMC4024371

[b16] Sabate R., Rousseau F., Schymkowitz J., Batlle C. & Ventura S. Amyloids or prions? That is the question. Prion 9, 200–206 (2015). 26039159 10.1080/19336896.2015.1053685 PMC4601216

[b17] Sabate R., Rousseau F., Schymkowitz J. & Ventura S. What makes a protein sequence a prion? PLoS Comput. Biol. 11, e1004013 (2015). 25569335 10.1371/journal.pcbi.1004013 PMC4288708

[b18] Rousseau F., Serrano L. & Schymkowitz J. W. How evolutionary pressure against protein aggregation shaped chaperone specificity. J. Mol. Biol. 355, 1037–1047 (2006). 16359707 10.1016/j.jmb.2005.11.035

[b19] Zambrano R. *et al.* PrionW: a server to identify proteins containing glutamine/asparagine rich prion-like domains and their amyloid cores. Nucleic Acids Res. 43, W331–W337 (2015). 25977297 10.1093/nar/gkv490 PMC4489250

[b20] Wan W. & Stubbs G. Fungal prion HET-s as a model for structural complexity and self-propagation in prions. Proc. Natl. Acad. Sci. USA 111, 5201–5206 (2014). 24706820 10.1073/pnas.1322933111 PMC3986130

[b21] Wickner R. B. [URE3] as an altered URE2 protein: evidence for a prion analog in Saccharomyces cerevisiae. Science 264, 566–569 (1994). 7909170 10.1126/science.7909170

[b22] Serio T. R. & Lindquist S. L. The yeast prion [PSI+]: molecular insights and functional consequences. Adv. Protein Chem. 59, 391–412 (2001). 11868278 10.1016/s0065-3233(01)59012-9

[b23] Du Z., Park K. W., Yu H., Fan Q. & Li L. Newly identified prion linked to the chromatin-remodeling factor Swi1 in Saccharomyces cerevisiae. Nat. Genet. 40, 460–465 (2008). 18362884 10.1038/ng.112 PMC2633598

[b24] Grishin A. V., Rothenberg M., Downs, & Blumer K. J. Mot3, a Zn finger transcription factor that modulates gene expression and attenuates mating pheromone signaling in Saccharomyces cerevisiae. Genetics 149, 879–892 (1998). 9611199 10.1093/genetics/149.2.879 PMC1460180

[b25] True H. L. & Lindquist S. L. A yeast prion provides a mechanism for genetic variation and phenotypic diversity. Nature 407, 477–483 (2000). 11028992 10.1038/35035005

[b26] Halfmann R. *et al.* Prions are a common mechanism for phenotypic inheritance in wild yeasts. Nature 482, 363–368 (2012). 22337056 10.1038/nature10875 PMC3319070

[b27] Travers A. A. The reprogramming of transcriptional competence. Cell 69, 573–575 (1992). 1316804 10.1016/0092-8674(92)90218-2

[b28] Magasanik B. The transduction of the nitrogen regulation signal in Saccharomyces cerevisiae. Proc. Natl. Acad. Sci. USA 102, 16537–16538 (2005). 16275904 10.1073/pnas.0507116102 PMC1283824

[b29] Fernandez-Escamilla A. M., Rousseau F., Schymkowitz J. & Serrano L. Prediction of sequence-dependent and mutational effects on the aggregation of peptides and proteins. Nat. Biotechnol. 22, 1302–1306 (2004). 15361882 10.1038/nbt1012

[b30] de Groot N. S., Castillo V., Grana-Montes R. & Ventura S. AGGRESCAN: method, application, and perspectives for drug design. Methods Mol. Biol. 819, 199–220 (2012). 22183539 10.1007/978-1-61779-465-0_14

[b31] Walsh I., Seno F., Tosatto S. C. & Trovato A. PASTA 2.0: an improved server for protein aggregation prediction. Nucleic Acids Res. 42, W301–W307 (2014). 24848016 10.1093/nar/gku399 PMC4086119

[b32] Nelson R. *et al.* Structure of the cross-beta spine of amyloid-like fibrils. Nature 435, 773–778 (2005). 15944695 10.1038/nature03680 PMC1479801

[b33] Sarroukh R., Goormaghtigh E., Ruysschaert J. M. & Raussens V. ATR-FTIR: a “rejuvenated” tool to investigate amyloid proteins. Biochim. Biophys. Acta 1828, 2328–2338 (2013). 23746423 10.1016/j.bbamem.2013.04.012

[b34] LeVine H. 3rd. Thioflavine T interaction with synthetic Alzheimer’s disease beta-amyloid peptides: detection of amyloid aggregation in solution. Protein Sci. 2, 404–410 (1993). 8453378 10.1002/pro.5560020312 PMC2142377

[b35] Sabate R., Rodriguez-Santiago L., Sodupe M., Saupe S. J. & Ventura S. Thioflavin-T excimer formation upon interaction with amyloid fibers. Chem. Commun. (Camb.) 49, 5745–5747 (2013). 23687656 10.1039/c3cc42040j

[b36] Ter-Avanesyan M. D., Dagkesamanskaya A. R., Kushnirov V. V. & Smirnov V. N. The SUP35 omnipotent suppressor gene is involved in the maintenance of the non-Mendelian determinant [psi+] in the yeast Saccharomyces cerevisiae. Genetics 137, 671–676 (1994). 8088512 10.1093/genetics/137.3.671 PMC1206026

[b37] Glover J. R. *et al.* Self-seeded fibers formed by Sup35, the protein determinant of [PSI+], a heritable prion-like factor of S. cerevisiae. Cell 89, 811–819 (1997). 9182769 10.1016/s0092-8674(00)80264-0

[b38] Liu J. J., Sondheimer N. & Lindquist S. L. Changes in the middle region of Sup35 profoundly alter the nature of epigenetic inheritance for the yeast prion [PSI+]. Proc. Natl. Acad. Sci. USA 99 Suppl 4, 16446–16453 (2002). 12461168 10.1073/pnas.252652099 PMC139907

[b39] Morris A. M., Watzky M. A. & Finke R. G. Protein aggregation kinetics, mechanism, and curve-fitting: a review of the literature. Biochim. Biophys. Acta 1794, 375–397 (2009). 19071235 10.1016/j.bbapap.2008.10.016

[b40] Tanaka M., Collins S. R., Toyama B. H. & Weissman J. S. The physical basis of how prion conformations determine strain phenotypes. Nature 442, 585–589 (2006). 16810177 10.1038/nature04922

[b41] Serio T. R. & Lindquist S. L. The yeast prion [PSI+]: molecular insights and functional consequences. Adv. Protein Chem. 59, 391–412 (2001). 11868278 10.1016/s0065-3233(01)59012-9

[b42] De Baets G., Van Doorn L., Rousseau F. & Schymkowitz J. Increased Aggregation Is More Frequently Associated to Human Disease-Associated Mutations Than to Neutral Polymorphisms. PLoS Comput. Biol. 11, e1004374 (2015). 26340370 10.1371/journal.pcbi.1004374 PMC4560525

[b43] de Groot N., Pallares I., Aviles F., Vendrell J. & Ventura S. Prediction of “hot spots” of aggregation in disease-linked polypeptides. BMC Struct. Biol. 5, 18 (2005). 16197548 10.1186/1472-6807-5-18 PMC1262731

[b44] Kim H. J. *et al.* Mutations in prion-like domains in hnRNPA2B1 and hnRNPA1 cause multisystem proteinopathy and ALS. Nature 495, 467–473 (2013). 23455423 10.1038/nature11922 PMC3756911

[b45] Paul K. R., Hendrich C. G., Waechter A., Harman M. R. & Ross E. D. Generating new prions by targeted mutation or segment duplication. Proc. Natl. Acad. Sci. USA 112, 8584–8589 (2015). 26100899 10.1073/pnas.1501072112 PMC4507246

[b46] Ventura S. *et al.* Short amino acid stretches can mediate amyloid formation in globular proteins: the Src homology 3 (SH3) case. Proc. Natl. Acad. Sci. USA 101, 7258–7263 (2004). 15123800 10.1073/pnas.0308249101 PMC409906

[b47] Ivanova M. I., Sawaya M. R., Gingery M., Attinger A. & Eisenberg D. An amyloid-forming segment of {beta}2-microglobulin suggests a molecular model for the fibril. PNAS %R 10.1073/pnas.0403756101 101, 10584–10589 (2004). PMC489978 15249659

[b48] Esteras-Chopo A., Serrano L. & de la Paz M. L. The amyloid stretch hypothesis: Recruiting proteins toward the dark side. PNAS %R 10.1073/pnas.0505905102 102, 16672–16677 (2005). PMC1283810 16263932

[b49] Castillo V., Grana-Montes R., Sabate R. & Ventura S. Prediction of the aggregation propensity of proteins from the primary sequence: aggregation properties of proteomes. Biotechnol. J. 6, 674–685 (2011). 21538897 10.1002/biot.201000331

[b50] Dovidchenko N. V. & Galzitskaya O. V. Computational Approaches to Identification of Aggregation Sites and the Mechanism of Amyloid Growth. Adv. Exp. Med. Biol. 855, 213–239 (2015). 26149932 10.1007/978-3-319-17344-3_9

[b51] Wang Q., Yu X., Li L. & Zheng J. Inhibition of amyloid-beta aggregation in Alzheimer’s disease. Curr. Pharm. Des. 20, 1223–1243 (2014). 23713775 10.2174/13816128113199990068

[b52] de Groot N. S., Aviles F. X., Vendrell J. & Ventura S. Mutagenesis of the central hydrophobic cluster in Abeta42 Alzheimer’s peptide. Side-chain properties correlate with aggregation propensities. FEBS J. 273, 658–668 (2006). 16420488 10.1111/j.1742-4658.2005.05102.x

[b53] Sabate R. *et al.* The role of protein sequence and amino acid composition in amyloid formation: scrambling and backward reading of IAPP amyloid fibrils. J. Mol. Biol. 404, 337–352 (2010). 20887731 10.1016/j.jmb.2010.09.052

[b54] Krebs M. R., Morozova-Roche L. A., Daniel K., Robinson C. V. & Dobson C. M. Observation of sequence specificity in the seeding of protein amyloid fibrils. Protein Sci. 13, 1933–1938 (2004). 15215533 10.1110/ps.04707004 PMC2279934

[b55] Toombs J. A. *et al.* *De novo* design of synthetic prion domains. Proc. Natl. Acad. Sci. USA 109, 6519–6524 (2012). 22474356 10.1073/pnas.1119366109 PMC3340034

[b56] Meszaros B., Dosztanyi Z. & Simon I. Disordered binding regions and linear motifs–bridging the gap between two models of molecular recognition. PLoS One 7, e46829 (2012). 23056474 10.1371/journal.pone.0046829 PMC3463566

[b57] Buck P. M., Kumar S. & Singh S. K. On the role of aggregation prone regions in protein evolution, stability, and enzymatic catalysis: insights from diverse analyses. PLoS Comput. Biol. 9, e1003291 (2013). 24146608 10.1371/journal.pcbi.1003291 PMC3798281

[b58] Uversky V. N. Intrinsically disordered proteins and their (disordered) proteomes in neurodegenerative disorders. Front Aging Neurosci 7, 18 (2015). 25784874 10.3389/fnagi.2015.00018 PMC4345837

[b59] Malinovska L., Kroschwald S. & Alberti S. Protein disorder, prion propensities, and self-organizing macromolecular collectives. Biochim. Biophys. Acta 1834, 918–931 (2013). 23328411 10.1016/j.bbapap.2013.01.003

[b60] Morris K. L. & Serpell L. C. X-ray fibre diffraction studies of amyloid fibrils. Methods Mol. Biol. 849, 121–135 (2012). 22528087 10.1007/978-1-61779-551-0_9

[b61] Collaborative Computational Project, N. The CCP4 suite: programs for protein crystallography. Acta Crystallogr. D Biol. Crystallogr. 50, 760–763 (1994). 15299374 10.1107/S0907444994003112

[b62] Makin O. S., Sikorski P. & Serpell L. C. CLEARER: a new tool for the analysis of X-ray fibre diffraction patterns and diffraction simulation from atomic structural models. J. Appl. Crystallogr. 40, 966–972 (2007).

[b63] Sabate R., Villar-Pique A., Espargaro A. & Ventura S. Temperature dependence of the aggregation kinetics of sup35 and ure2p yeast prions. Biomacromolecules 13, 474–483 (2012). 22176525 10.1021/bm201527m

[b64] Tanaka M., Chien P., Naber N., Cooke R. & Weissman J. S. Conformational variations in an infectious protein determine prion strain differences. Nature 428, 323–328 (2004). 15029196 10.1038/nature02392

