# Supplementary material for: Characterization of Amyloid Cores in Prion Domains
Source: Sci Rep. 2016 Sep 30;6:34274. doi: 10.1038/srep34274 (PMC5043269; doi:10.1038/srep34274)

Scientific Reports

Characterization of Amyloid Cores in Prion Domains

Ricardo Sant'Anna[1], Maria Rosario Fernandez[1], Cristina Batlle[1], Susanna Navarro[1], Natalia S. de Groot[1], Louise Serpell[2] and Salvador Ventura[1]

[1]Institut de Biotecnologia i Biomedicina and Departament de Bioquimica i Biologia Molecular, Universitat Autònoma de Barcelona, 08193 Bellaterra, Barcelona, Spain.

[2] School of Life Sciences, University of Sussex, Falmer, BN1 9QG, UK

SUPPLEMENTARY INFORMATION:

Supplementary Table S1. X-ray diffraction signal positions

Supplementary Figure S1. Aggregation kinetics, seeding and cross-seeding reactions of Sup35 PFD amyloid core.

Table S1. X-ray diffraction signal positions

Diffraction signals (Å)	Swi1	Sup35	Ure2p	Mot3
Meridionals	4.70	4.75	4.68	4.65
Equatorials	18	21	23	17-26
		13		
	9	9.7	9	8.5
		7.4	6	

Figure S1. Aggregation kinetics, seeding and cross-seeding reactions of Sup35 PFD amyloid core. The aggregation kinetics were carried out by incubating 100µM of peptide with 30µM of Th-T at RT with no agitation and the fluorescence measured along time in the absence (no seed) or presence of 2 % of the fibrils formed by the four PFD peptides in the present study. Th-T spectra were collected by exciting the samples at 450 nm and collecting the emission from 460 to 600 nm. The intensity at 482 nm was used to monitor the extent of aggregation.

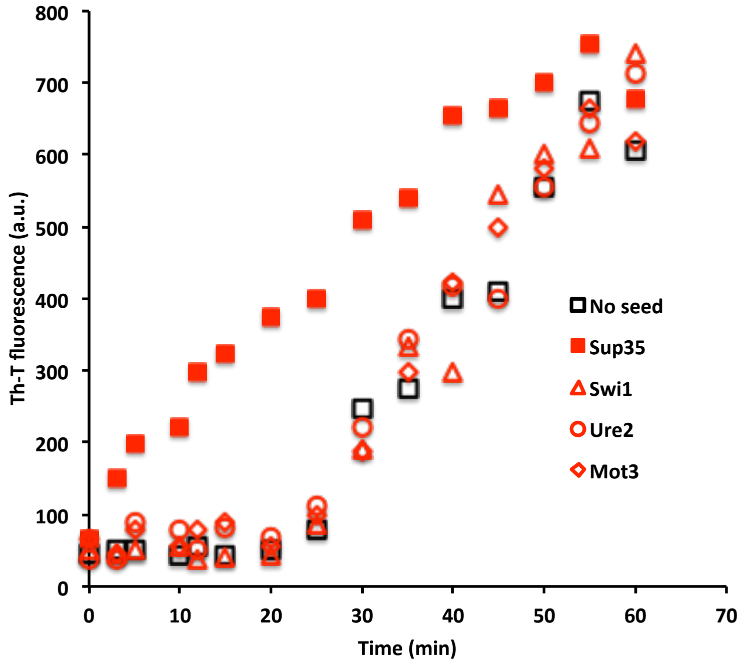

Supplement: Supplementary Information [file srep34274-s1.pdf]
